# The use, safety, and effectiveness of herpes zoster vaccination in individuals with inflammatory and autoimmune diseases: a longitudinal observational study

**DOI:** 10.1186/ar3497

**Published:** 2011-10-24

**Authors:** Jie Zhang, Elizabeth Delzell, Fenglong Xie, John W Baddley, Claire Spettell, Raechele M Mcmahan, Joaquim Fernandes, Lang Chen, Kevin Winthrop, Jeffrey R Curtis

**Affiliations:** 1 Department of Epidemiology, School of Public Health, University of Alabama at Birmingham, 1665 University Blvd, Birmingham, AL 35294, USA; 2 Division of Clinical Immunology and Rheumatology, School of Medicine, University of Alabama at Birmingham, 510 20th Street South, Birmingham, AL 35294, USA; 3 Division of Infectious Diseases, Department of Medicine, University of Alabama at Birmingham and Birmingham Veteran Affairs Medical Center, 1900 University Blvd., Birmingham, AL 35294, USA; 4 Aetna Informatics, Aetna, 980 Jolly Road, Blue Bell, PA 19422, USA; 5 Aetna Specialty Pharmacy, 503 Sunport Lane, Orlando, FL 32809, USA; 6 Division of Infectious Diseases, Department of Medicine, Oregon Health and Science University, 3181 SW Sam Jackson Park Road L457, Portland, OR 97239, USA

## Abstract

**Introduction:**

Zostavax, a live attenuated vaccine, has been approved in the United States for use in older individuals to reduce the risk and severity of herpes zoster (HZ), also known as shingles. The vaccine is contraindicated in individuals taking anti-tumor necrosis factor alpha (anti-TNF) therapies or other biologics commonly used to treat autoimmune diseases because of the safety concern that zoster vaccine may be associated with a short-term HZ risk. The objective of the study was to examine the use, safety (short-term HZ risk after vaccination), and effectiveness of zoster vaccine in individuals with rheumatoid arthritis, psoriasis, psoriatic arthritis, ankylosing spondylitis, and/or inflammatory bowel diseases.

**Methods:**

We conducted a cohort study of patients aged 50 years and older with rheumatoid arthritis, psoriasis, psoriatic arthritis, ankylosing spondylitis, and/or inflammatory bowel diseases by using administrative claims data from a nationwide health plan from January 1, 2005, to August 31, 2009. We examined the extent to which zoster vaccine was used; assessed factors associated with vaccine use (Cox proportional hazards regression); and compared the incidence rates of herpes zoster (HZ) between vaccinated and unvaccinated patients.

**Results:**

Among 44,115 patients with the autoimmune diseases, 551 (1.2%) received zoster vaccine, and 761 developed HZ. Zoster vaccine use increased continuously after approval in 2006. Younger and healthier patients, those who had an HZ infection within the past 6 months, and those who were not using anti-TNF therapies were more likely to receive the vaccine. Approximately 6% of vaccinated patients were using anti-TNF therapies at the time of vaccination. The incidence rates of HZ were similar in vaccinated and unvaccinated patients (standardized incidence ratio, 0.99; 95% confidence interval, 0.29 to 3.43).

**Conclusions:**

Use of the zoster vaccine was uncommon among older patients with autoimmune diseases, including those not exposed to immunosuppressive medications. The short-term risk of HZ did not appear to be increased in vaccinated patients, even among those using immunosuppressive therapies (for example, biologics) at the time of vaccination. However, our study was limited by the small number of vaccinated patients, and further evidence is needed to confirm the vaccine's safety and efficacy in this population.

## Introduction

Herpes zoster (HZ) infection, also known as shingles, is caused by the reactivation of latent varicella-zoster virus (VZV) and usually occurs decades after primary infection. HZ is characterized by a painful blistering rash and occurs mostly in older adults. Many patients experience persistent pain after the rash heals, a common yet debilitating complication known as postherpetic neuralgia (PHN) [1,2]. In the United States, the age- and sex-adjusted incidence rate (IR) of HZ is estimated to be 3.0 to 4.0 per 1,000 person-years [3-5]. Older age is the most important risk factor for the development of both HZ and PHN [3,4]. Disease risk is elevated in individuals who are immune-suppressed due to human immunodeficiency virus (HIV) infection and transplantation [3,4,6]. Recent studies reported an increased risk of HZ in patients with rheumatoid arthritis (RA) that is attributable both to the disease and to treatment with anti-tumor necrosis factor alpha (anti-TNF) therapies and other immunosuppressive agents [7,8]. In addition, anti-TNF therapies has been associated with a more severe course of HZ among patients with rheumatic diseases; those receiving anti-TNF therapies were 9 times more likely than those not to be hospitalized for HZ [9].

Zostavax, a live attenuated vaccine, was approved in 2006 for use in individuals 60 years of age or older to reduce the risk and severity of HZ. The Advisory Committee on Immunization Practices (ACIP) recommended that all adults older than 60 years be considered for vaccination, with certain exceptions [10]. Along with patients with certain malignancies and HIV/AIDS, patients receiving biologic agents such as anti-TNF therapies, some nonbiologic disease-modifying antirheumatic drugs (DMARDs), such as high doses of methotrexate, and high doses of glucocorticoids were also excluded. The concern is that zoster vaccine, a live vaccine, could acutely trigger the development of HZ in patients with compromised immune systems in a short time frame (for example, within 4 to 6 weeks after vaccination) despite conferring a longer-term protection against the development of HZ. Based on expert opinion, glucocorticoids at prednisone-equivalent doses up to 20 mg/day, low-doses of methotrexate (< 0.4 mg/kg/week, a typical dose for patients with autoimmune diseases), azathioprine (< 3.0 mg/kg/day), and 6-mercaptopurine (< 1.5 mg/kg/day) were considered acceptably safe so as not to require a contraindication for use of zoster vaccine. Similarly, the American College of Rheumatology (ACR) 2008 guidelines for the use of biologic and nonbiologic DMARDS did not recommend the administration of zoster vaccine to patients with RA or other rheumatic diseases treated with biologics [11].

In light of the uncertainties regarding the use, safety, and effectiveness of zoster vaccine in this population, we used the administrative claims data of a large nationwide health plan to investigate the following aims in a cohort of patients diagnosed with RA, psoriatic arthritis, psoriasis, ankylosing spondylitis, and/or inflammatory bowel diseases: (a) to describe the use of zoster vaccination over time; (b) to determine the extent to which zoster vaccine was used in patients taking concomitant immunosuppressive agents; (c) to assess patient characteristics associated with receipt of zoster vaccine; and (d) to evaluate the incidence of HZ in patients who received and did not receive zoster vaccine.

## Materials and methods

### Study population

We conducted a retrospective cohort study by using administrative claims data from Aetna, a nationwide health plan that provides medical coverage to more than 17 million individuals in the United States, from January 1, 2006, to August 31, 2009. The cohort included patients with RA, psoriatic arthritis, psoriasis, ankylosing spondylitis, and inflammatory bowel diseases, identified by using the International Classification of Diseases, 9th revision (ICD-9) and National Drug Codes (NDC) codes. Patients were included if there were (a) two ICD-9 diagnosis codes from an outpatient physician or hospital encounter, separated by at least 7 days and occurring within 365 days; or (b) one physician- or hospital-encounter diagnosis code followed by a prescription for medications used to treat autoimmune diseases within 365 days (diagnosis codes and medications listed in Additional file 1). We defined each subject's index date (patients consist of a mix of incident and prevalent cases) as the date of the second diagnosis code or the date when the prescription was filled. Patients who met definitions for two or more of the diseases of interest were categorized into a multiple diseases category, and their index date was defined as the earliest of all disease-specific index dates.

To ensure that we had complete medical and pharmacy claims necessary to identify and characterize the study population, all subjects were required to have a "baseline period" of at least 183 continuous days during which they had medical and pharmacy benefits; subjects aged 65 or older had to be enrolled in a Medicare Advantage plan with a concomitant pharmacy benefit administered by Aetna. Follow-up started on the later of the index date or the date on which the subject had a baseline period of at least 183 days. Subjects were censored when they lost either medical or pharmacy benefits, died, or for individuals aged 65 years or older, if Aetna became the secondary payer to Medicare. Patients were excluded if they were younger than 50 years at the start of follow-up or received zoster vaccine during the baseline period. Although the ACIP recommended the use of zoster vaccine in immune-competent individuals aged 60 years or older [10], given the increased HZ risk previously observed in this population, we included those between 50 and 60 years of age in our analysis to assess the extent to which zoster vaccine was used.

### Assessment of vaccination status, HZ infection, and exposure to immunosuppressive agents

Zoster vaccine was covered under the health plan's medical benefit for individuals aged 60 or older beginning June 1, 2006, and was ascertained by using the Current Procedural Terminology (CPT) code 90736. Reimbursement of zoster vaccine for patients younger than 60 years was made on a case-by-case basis. Cases of HZ were identified by the first HZ claim for each patient that was preceded or followed by a prescription for acyclovir, famciclovir, and valacyclovir within 30 days of the HZ claim date. Exposures to immunosuppressive agents were ascertained by the days' supply for all relevant medications. To estimate cumulative glucocorticoid exposure as a time-varying variable, cumulative average daily doses were calculated by summing the total amount of oral glucocorticoids prescribed in the preceding 6 months and then dividing the total amount by 183 days. Subjects were then categorized into four groups based on their cumulative average daily prednisone-equivalent glucocorticoid dose: none, low (less than 10 mg/day), medium (10 to 20 mg/day), and high (greater than 20 mg/day).

### Statistical analyses

To characterize use of the zoster vaccination (Aim 1), we calculated the incidence proportion of zoster vaccination for each 6-month interval beginning July 1, 2006, and ending August 31, 2009. For each time interval, the denominator was the number of subjects who were unvaccinated at the start of the interval and were under observation throughout the entire interval, and the numerator was the number of these subjects who were vaccinated during the interval. Because we had claims up to August 31, 2009, the incidence proportion from July 1, 2009, to August 31, 2009, was multiplied by 3 to approximate the incidence proportion for the 6-month period ending December 31, 2009.

To characterize the immune status of patients at time of vaccination (Aim 2), we described the numbers and percentages of patients who used anti-TNF therapies (etanercept, infliximab, adalimumab), other biologic agents (abatacept, rituximab), traditional DMARDs (methotrexate, hydroxychloroquine, sulfasalazine, azathioprine, leflunomide, mercaptopurine), and oral glucocorticoids 30 days before and up to 30 days after the administration of zoster vaccine. These analyses were conducted in patients who received zoster vaccine and were followed up for at least 30 days after vaccination.

For Aim 3, we used Cox proportional hazards regression models to assess patient characteristics associated with time to HZ vaccination. Subject characteristics examined were gender, type of autoimmune disease, and several time-varying factors including age, co-morbidities measured by using the Charlson co-morbidity index [12], number of physician visits, hospitalization (yes or no), history of HZ infection (recent, within the past 6 months; remote, more than 6 months ago), history of influenza and pneumococcal vaccine (within the past 12 months), and use of medications listed in Aim 2. Whenever an event occurred, the time-varying characteristics of interest were ascertained as of that time for all patients by using claims in the preceding 6-months period, unless otherwise specified.

For Aim 4, accrual of person-time began at the start of follow-up, and a vaccinated subject would contribute both unvaccinated and vaccinated person-time. We calculated the crude and age- and sex-specific incidence rates of HZ in vaccinated and unvaccinated person-times. We applied the age- and sex-specific incidence rates of the unvaccinated to age- and sex-specific vaccinated person-time to derive the expected number of HZ cases among the vaccinated and calculated the standardized incidence-rate ratio as the observed divided by the expected number of cases [13].

### Sensitivity analyses

Because zoster vaccine was approved for use in the United States in May 2006 in individuals aged 60 or older, sensitivity analyses were conducted (a) in patients for whom we had complete medical and pharmacy claims prior to or since June 1, 2006, to avoid misclassification of vaccination status in patients who might have been vaccinated before joining the health plan; and (b) in patients aged 60 or older as of the start of follow-up. Because HZ infections diagnosed late in the course of the infection might not benefit from or receive antiviral medications, and approximately 10% to 20% of HZ cases were not treated with antiviral medication, we conducted a sensitivity analysis that used an alternate definition for HZ that required only the HZ diagnosis code to calculate incidences of HZ in vaccinated and unvaccinated patients.

The University of Alabama at Birmingham Institutional Review Board approved the study and waiver of informed consent.

## Results

**Figure 1:**
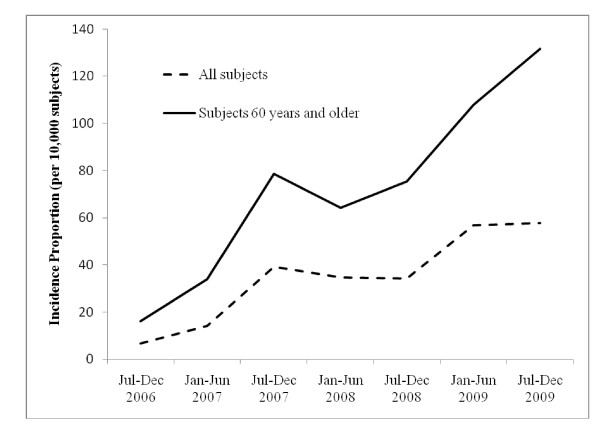
**Incidence proportion for each 6-month period of zoster vaccination by calendar year from June 1, 2006, to December 31, 2009**. The proportion is expressed as the proportion of unvaccinated individuals under observation and vaccinated in each 6-month period. It does not include individuals vaccinated in previous time periods.

We identified 44,115 eligible subjects classified as having at least one of the autoimmune diseases of interest who were at least 50 years old at the start of observation. At baseline, their average age was 58.1 (standard deviation (SD), 6.7) years and 27,443 (62.2%) were female; 19,326 (43.8%) had RA, 867 (2.0%) had psoriatic arthritis (PsA), 10,712 (24.3%) had psoriasis (PsO), 633 (1.4%) had AS, 8,639 (19.6%) had IBD, and 3,938 (8.9%) had two or more of these diseases. During follow-up, 551 (1.2%) subjects received zoster vaccine. The earliest claim for vaccination occurred in August 2006; subsequently, vaccine use increased continuously over time (Figure 1). The distribution of patients' characteristics at baseline by vaccination status is presented in Table 1.

**Table 1:** Baseline patient characteristics by vaccination status

	Vaccination status	
Baseline characteristics, *n *(%)	Vaccinated *n *= 551	Unvaccinated *n *= 43,564
Age groups (years)		
50 to 59	202 (36.7)	30,156 (69.2)
60 to 64	275 (49.9)	9,573 (22.0)
65 and older	74 (13.4)	3,835 (8.8)
Women	352 (63.9)	27,091 (62.2)
Inflammatory/Autoimmune disease		
RA	206 (37.4)	19,120 (43.9)
Psoriatic arthritis	11 (2.0)	856 (2.0)
Psoriasis	146 (26.5)	10,566 (24.3)
Inflammatory bowel diseases	136 (24.7)	8,503 (19.5)
Ankylosing spondylitis	8 (1.5)	625 (1.4)
Multiple diseases	44 (8.0)	3,894 (8.9)
Medications^a^		
TNF antagonists user	27 (4.9)	4,186 (9.6)
Other biologics user	0 (0)	198 (0.5)
Conventional DMARDs user	92 (16.7)	9,312 (21.4)
Oral glucocorticoids^b^		
None	456 (82.8)	34,267 (78.7)
Low	86 (15.6)	8,041 (18.5)
Medium	7 (1.3)	902 (2.1)
High	2 (0.4)	354 (0.8)
Charlson comorbidity index		
0	287 (52.1)	19,971 (45.8)
1	181 (32.9)	16,565 (38.0)
≥ 2	83 (15.1)	7,028 (16.1)
Inpatient hospitalization		
No	466 (84.6)	36,158 (83.0)
Yes	45 (15.4)	7,406 (17.0)
Number of outpatient physician visits		
0-2	83 (15.1)	8,705 (20.0)
3-5	227 (41.2)	16,843 (38.7)
6-10	185 (33.6)	13,376 (30.7)
11 and more	54 (9.8)	4,642 (10.7)

DMARDS, Disease-modifying antirheumatic drugs; RA, rheumatoid arthritis; TNF, tumor necrosis factor. ^a^Measured in the 6-month baseline period before the start of observation

^b^Cumulative average daily prednisone-equivalent glucocorticoid dose in the past 183 days: none, low (< 10 mg/day), medium (10-20 mg/day), and high (> 20 mg/day).

Among the 551 subjects who received zoster vaccine, complete claims history for at least 30 days after vaccination was available for 514 subjects. At the time of vaccination, 32 (6.2%) subjects were using anti-TNF therapy, 34 (6.6%) were using methotrexate, and 33 (6.4%) were using oral glucocorticoids (Table 2). In the 30 days after vaccination, 40 (7.8%) subjects used anti-TNF therapy, 45 (8.8%) used methotrexate, and 48 (9.3%) used oral glucocorticoids. A total of 47 patients used biologics at some time within 30 days before and after vaccination. None of the 47 subjects developed HZ in the 30 days after vaccination. Their average age was 60 years (standard deviation, 5); 64% were women; and 70% were not exposed to oral glucocorticoids, 28% to a daily average dose of no more than 20 mg, and 2% to a daily average dose of 20 mg or more.

**Table 2:** Biologic and nonbiologic DMARDs use 30 days before and after zoster vaccination (*n *= 514)

	30 days before vaccination		Day of vaccination		30 days after vaccination	
	*n*	%	*n*	%	*n*	%
Anti-TNF	40	7.8	32	6.2	40	7.8
Etanercept	18	3.5	12	2.3	18	3.5
Adalimumab	11	2.1	9	1.8	11	2.1
Infliximab	11	2.1	11	2.1	11	2.1
Other biologics^a^	4	0.8	4	0.8	4	0.8
Nonbiologic DMARDs						
Methotrexate	52	10.1	34	6.6	45	8.8
Sulfasalazine	16	3.1	13	2.5	16	3.1
Hydroxychloroquine	26	5.1	17	3.3	25	4.9
Azathioprine	1	0.2	1	0.2	1	0.2
Leflunomide	3	0.6	3	0.6	3	0.6
Cyclosporine	7	1.4	6	1.2	7	1.4
6-Mercaptopurine	5	1.0	3	0.6	5	1.0
Oral glucocorticoid	45	8.8	33	6.4	48	9.3

DMARDS, Disease-modifying antirheumatic drugs; RA, rheumatoid arthritis; TNF, tumor necrosis factor. ^a^The categories (columns) are not exclusive (for example, a patient who used anti-TNF therapies continuously during the 60-day period would be counted in all three categories). ^b^Abatacept, rituximab.

Patients using anti-TNF therapies were less likely to receive zoster vaccine than those who were not using anti-TNF agents (hazard ratio (HR), 0.47; 95% CI, 0.33 to 0.67) (Table 3). Patients who were using other biologics (HR, 0.52; 95% CI, 0.19 to 1.40) or high-dose oral glucocorticoids (HR, 0.46; 95% CI, 0.15 to 1.45) were only half as likely as were non-users to receive zoster vaccine, but these associations did not reach statistical significance. Patients aged 60 to 64 years were most likely to receive zoster vaccine; those with fewer co-morbidities and those without hospitalization within the past 6 months were more likely to receive zoster vaccine. Patients with recent, but not remote, history of HZ were more likely to be vaccinated. Finally, patients who had more physician visits in outpatient settings and those who had received influenza or pneumococcal vaccine in the past year were more likely to be vaccinated. Results of the analysis that was restricted to individuals aged 60 years or older were similar to the main results (Table 3). A majority (> 80%) of the patients received their vaccine from family practice or internal medicine physicians, with less than 5% from dermatologists, gastroenterologists, or rheumatologists, both in the overall cohort and in patients who were exposed to biologics at the time of vaccination.

**Table 3:** Fixed and time-varying^a ^patient characteristics associated with vaccination

	All subjects		Subjects aged 60 years or older^b^	
Baseline	Hazard ratio	95% Confidence interval	Hazard ratio	95% Confidence interval
Age groups, years				
50-59	**0.06**	**0.05-0.08**	NA	NA
60-64^c^	1.00	**-**	**-**	**-**
≥ 65	**0.41**	**0.31-0.53**	**0.42**	**0.33-0.54**
Women, *n *(%)	1.13	0.95-1.35	1.17	0.97-1.41
Disease status				
Rheumatoid arthritis^d^	1.00	**-**	**-**	**-**
Psoriatic arthritis	1.58	0.86-2.91	1.27	0.63-2.59
Psoriasis	**1.48**	**1.17-1.86**	**1.54**	**1.21-1.95**
Inflammatory bowel diseases	**1.71**	**1.36-2.14**	**1.64**	**1.29-2.09**
Ankylosing spondylitis	1.45	0.71-2.97	1.23	0.54-2.79
Multiple diseases	1.00	0.72-1.39	1.01	0.71-1.43
History of herpes zoster infection				
None^e^	1.00	**-**	**-**	**-**
Recent	**2.84**	**1.34-6.01**	2.21	0.91-5.35
Remote	0.93	0.51-1.69	0.82	0.42-1.59
Medications (current use)				
TNF antagonists	**0.47**	**0.33-0.67**	**0.41**	**0.27-0.61**
Other biologics (see Additional file 1)	0.52	0.19-1.40	0.59	0.22-1.58
Conventional DMARDs	0.92	0.72-1.19	0.91	0.70-1.19
Oral glucocorticoids				
None^f^	1.00	**-**	**-**	**-**
Low/medium	0.84	0.66-1.08	0.85	0.66-1.10
High	0.46	0.15-1.45	0.53	0.17-1.68
Charlson co-morbidity index				
0^g^	1.00	**-**	**-**	**-**
1	**0.76**	**0.61-0.95**	**0.77**	**0.61-0.97**
≥ 2	**0.57**	**0.42-0.78**	**0.57**	**0.42-0.79**
One or more inpatient physician visit	**0.53**	**0.40-0.72**	**0.50**	**0.37-0.69**
Number of outpatient physician visits				
0-2^h^	1.00	**-**	**-**	**-**
3-5	**1.68**	**1.32-2.14**	**1.70**	**1.31-2.19**
6-10	**1.80**	**1.39-2.34**	**1.80**	**1.37-2.38**
> 10	**2.08**	**1.47-2.93**	**2.03**	**1.41-2.92**
Influenza vaccine in the past year	**2.25**	**1.87-2.71**	**2.05**	**1.69-2.49**
Pneumococcal vaccine in the past year	**1.79**	**1.45-2.21**	**1.81**	**1.45-2.26**

^a^Gender and disease status were fixed; all other characteristics were time-varying. Time-varying characteristics were ascertained by using claims in the preceding 6-month period, unless otherwise specified in the table. ^b^Sensitivity analysis restricted to individuals aged 60 and older.

Reference groups were as follows: ^c^age category 60-64; ^d^RA patients; ^e^Subjects without prior zoster infection; ^f^subjects not prescribed oral glucocorticoids in the past 6 months (cumulative average daily prednisone-equivalent glucocorticoid dose: low/medium ≤20 mg/day and high (> 20 mg/day); ^g^subjects with Charlson score = 0; ^h^subject who had none to two outpatient physician encounter (s) in the past 6 months. DMARDS, disease-modifying antirheumatic drugs; RA, rheumatoid arthritis; TNF, tumor necrosis factor. Bolded data indicates statistically significant associations.

During 88,354 observed person-years, 761 cases of HZ occurred (incidence rate (IR), 8.6 per 1,000 person-years). The IR increased with age from 8.14 per 1,000 person-years among those aged 50 to 54 years to 15.30 per 1,000 person-years among those 90 years or older. Five cases of HZ occurred during vaccinated person-time (crude IR, 9.97 per 1,000 person-years), and 756 HR occurred during unvaccinated person-time (crude IR, 8.61 per 1,000 person-years). The age- and sex-standardized IR (expected IR) for the vaccinated was 10.06 per 1,000 person-years, resulting in a standardized IR ratio (vaccinated to unvaccinated) of 0.99 (95% CI, 0.29 to 3.43).

The five cases of HZ in the vaccinated patients occurred 7, 131, 201, 214, and 667 days after vaccination. Within 90 days before vaccination, the only filled prescription for an immunosuppressive agent for the patient who developed HZ on day 7 after vaccination was for a 15-day supply of prednisone approximately 2 months before receipt of zoster vaccination. None of the five patients was hospitalized.

Results from sensitivity analyses restricted to individuals under observation continuously from the time zoster vaccine was approved in 2006 had similar results (data not shown). When requiring only an HZ diagnosis code, the result was consistent, in that the crude incidence rates were the same between vaccinated (14.4 per 1,000 person-years) and unvaccinated (13.1 per 1,000 person-years) patients. With Poisson regression, the incidence rate ratio was 0.99 (95% CI, 0.35 to 2.82).

## Discussion

In this large prospective cohort study of more than 40,000 patients with autoimmune diseases, we showed that despite their increased risk of developing HZ, only 551 (1.2%) received zoster vaccine. We also showed that approximately 6% of those who received the vaccine were currently using anti-TNF therapies, and none developed HZ within 1 month after vaccination.

The administration of zoster vaccine in patients exposed to biologics is in conflict with recommendations from ACIP and ACR. More than 80% of the vaccinated patients received their vaccine from their primary care physicians; it is possible that they may not be aware of their patients' exposure to immunosuppressive agents or the contraindication. In patients with immune-mediated inflammatory diseases who receive targeted immunosuppressive therapies (for example, anti-TNF therapy), it is currently not known whether the benefits of zoster vaccine outweigh any theoretic safety concerns, and many experts have called for studies to evaluate the safety and effectiveness of zoster vaccine in this patient population [14,15]. In pediatric HIV patients, varicella vaccine has been administered safely in children with or without primary varicella infection [16,17] and is highly effective in preventing varicella infection and subsequent HZ [18]. Moreover, yellow-fever vaccine, another live attenuated vaccine, was administered to 17 RA patients who were previously immunized and were currently receiving infliximab; none reported yellow-fever related symptoms after vaccination [19].

In our study, among 551 vaccinated subjects, HZ developed in five after vaccination; none was hospitalized, and one occurred within 1 month of vaccination. Of interest, the patient in whom HZ developed within 1 month after vaccination was not exposed to biologics, glucocorticoids, or traditional DMARDs at time of vaccination. In the Shingles Prevention Study, seven confirmed cases of HZ occurred within 42 days after vaccination among 19,270 vaccinated subjects [20]. We observed only one such case among 551 vaccinated patients with autoimmune diseases. Taken together, our result suggests that a short-term increase in the risk of infection might not exist, as might be feared with a live-virus vaccine. However, although our results did not raise any safety concern, it is important to note that the administration of zoster vaccine to these individuals was selective. Subjects who took biologics and high doses of oral glucocorticoids were less likely to be immunized, whereas younger and healthier patients were more likely to be vaccinated. Our study suggested that zoster vaccine can be administered safely to a selected subgroup of patients with the autoimmune diseases studied but did not provide definitive evidence that it is safe for all.

In considering the effectiveness of the zoster vaccine in this population, we did not find any difference in the incidence rates of HZ among the vaccinated and the unvaccinated patients. However, this and other findings must be interpreted in light of the study' limitations. The most important limitation is the small number of patients who received zoster vaccine (*n *= 551) and the even fewer in whom HZ developed after vaccination (*n *= 5). As a result, the overall IRs, and especially the age- and sex-specific IRs, were not so reliable. Coupled with a selection bias that those who were vaccinated were younger, healthier, and less likely to be immune suppressed, no conclusion could be drawn from the finding that incidence rates of HZ in vaccinated and unvaccinated patients were comparable. Misclassification of immunosuppressive agent use might have occurred if patients filled prescriptions from a pharmacy, but they were verbally told by their healthcare provider to discontinue the medications temporarily before vaccination. Another limitation of the study is that we lacked medical records to confirm or evaluate the severity of HZ cases identified by using the claims data. Finally, we did not have information on race/ethnicity, a factor that has been associated with risk of HZ [21].

The underuse of zoster vaccine concerns not only patients with contraindications but also those who had no contraindication to the use of zoster vaccine. One of the barriers is likely provider concern about inadvertent administration of the vaccine to immune-suppressed patients [22]. Another possible reason is the lack of information on the efficacy and safety of zoster vaccine in these patients, who were not included in vaccine clinical trials. In addition, we found that older patients were less likely to receive zoster vaccine despite the ACIP recommendations supporting its use among individuals older than 60 years. This finding is consistent with those from previous studies that showed immunization lagging in older adults [23] and raises a serious concern because zoster vaccine appears to be underused in the population with the highest disease risk. A number of barriers to the use of zoster vaccine have been identified, with the most important being financial concerns, reimbursement issues, and storage difficulties [22]. In our study, zoster vaccine use increased continuously since it was approved in 2006. This points to another potential barrier to the use of the zoster vaccine (the newness of the vaccine) and suggests that programs educating primary care physicians and specialists may help to increase the appropriate vaccine use. As expected, more frequent physician contact and receipt of other vaccinations, including influenza or pneumococcal vaccine, was associated with a greater likelihood of receiving zoster vaccine. These associations may reflect physician attentiveness and patients' health-seeking behaviors.

## Conclusions

Zoster vaccine was underused in patients with autoimmune diseases despite their increased risk of developing HZ. Contrary to clinical guidelines, a small number of patients receiving anti-TNF therapies were vaccinated, and none developed zoster infection within 30 days after vaccination. The incidence rates of HZ were similar in the vaccinated and unvaccinated person-times. The results regarding safety and effectiveness are preliminary, and future evaluations are needed to understand better the risks and benefits associated with zoster vaccine in patients with autoimmune disease and to help refine guidelines for the use of zoster vaccine.

## Abbreviations

ACIP: the Advisory Committee on Immunization Practices; ACR: American College of Rheumatology; anti-TNF: anti-tumor necrosis factor alpha; CPT: current procedural terminology; DMARDs: disease-modifying antirheumatic drugs; HIV: human immunodeficiency virus; HZ: hazard ratio; HZ: herpes zoster; ICD-9: International Classification of Diseases, 9^th ^edition; IR: incidence rate; NDC: national drug code; PHN: postherpetic neuralgia; RA: rheumatoid arthritis; SD: standard deviation; VZV: varicella zoster virus.

## Competing interests

ED receives research funding from Amgen; JWB is a Board Member of Merck, is a consultant to Pfizer, and receives research funding from Pfizer; RMM's institution receives funding from Centocor, Bristol Myers Squibb, and Abbott; JF receives a salary from Aetna; and JRC has received research grants and consulting fees from Amgen, UCB, Abbott, Genentech, Roche, Centocor, BMS, and Merck.

## Authors' contributions

JRC conceived the research questions and hypotheses, refined the research questions and hypotheses, planned the statistical analyses, and gave significant input to the draft of the manuscript with regard to the interpretation of results. JZ conceived the research questions and hypotheses, refined the research questions and hypotheses, planned the statistical analyses, and drafted the manuscript. ESD and JWB conceived the research questions and hypotheses, refined the research questions and hypotheses, and gave significant input to the draft of the manuscript with regard to the interpretation of results. KW gave significant input to the draft of the manuscript with regard to the interpretation of results. FX refined the research questions and hypotheses, planned and performed statistical analyses, and gave significant input to the draft of the manuscript with regard to the interpretation of results. LC refined the research questions and hypotheses and planned and performed statistical analyses. CS, RMM, and JF assisted in acquiring data. ED planned the statistical analyses. All authors critically reviewed and approved the final manuscript for publication.

## Acknowledgements

This work was supported by the Agency for Healthcare Research and Quality (R01HS018517) and the Doris Duke Charitable Foundation. JRC received support from the National Institutes of Health (AR053351); JZ received support from the Agency for Healthcare Research and Quality (T32HS013852).

## Supplementary Material

Additional file 1 **ICD9-diagnosis codes and medications used in case definitions**. Click here for file
