# Supplementary material for: The use, safety, and effectiveness of herpes zoster vaccination in individuals with inflammatory and autoimmune diseases: a longitudinal observational study
Source: Arthritis Res Ther. 2011 Oct 24;13(5):R174. doi: 10.1186/ar3497 (PMC3308109; doi:10.1186/ar3497)
Supplement: Additional file 1 — ICD9-diagnosis codes and medications used in case definitions. [file ar3497-S1.DOC]

**Appendix** ICD9-diagnosis codes and medications used in case definitions

|  | **RA** | **PsA** | **PS** | **AS** | **IBD** |
| --- | --- | --- | --- | --- | --- |
| **Medications** |  |  |  |  |  |
| Traditional DMARDs |  |  |  |  |  |
| Methotrexate | X | X | X | X | X |
| Hydroxychloroquine | X |  |  |  |  |
| Sulfasalazine | X | X |  | X | X |
| Azathioprine |  |  |  |  | X |
| Leflunomide | X | X |  | X |  |
| Cyclosporine |  |  | X |  |  |
| Acitretin |  |  | X |  |  |
| 6-Mercaptopurine |  |  |  |  | X |
|  |  |  |  |  |  |
| TNF antagonists |  |  |  |  |  |
| Etanercept | X | X | X | X | X |
| Infliximab | X | X | X | X | X |
| Adalimumab | X | X | X | X | X |
| Golimumab | X | X | X | X | X |
| Certolizumab | X |  |  |  | X |
| Natalizumab |  |  |  |  | X |
|  |  |  |  |  |  |
| Other biologics |  |  |  |  |  |
| Anakinra | X |  |  |  |  |
| Abatacept | X |  |  |  |  |
| Rituximab | X |  |  |  |  |
| Alefacept |  |  | X |  |  |
| Efalizumab (withdrawn) |  |  | X |  |  |
|  |  |  |  |  |  |
| Phototherapy |  |  | X |  |  |
|  |  |  |  |  |  |
| ICD-9 codes | 714.X | 696.0 | 696.1 | 720.0 | 555.x, 556.x |
